# Cis-Regulatory Mechanisms for Robust Olfactory Sensory Neuron Class-restricted Odorant Receptor Gene Expression in *Drosophila*

**DOI:** 10.1371/journal.pgen.1005051

**Published:** 2015-03-11

**Authors:** Shadi Jafari, Mattias Alenius

**Affiliations:** Department of Clinical and Experimental Medicine, Linköping University, Linköping, Sweden; K.U.Leuven, BELGIUM

## Abstract

Odor perception requires that each olfactory sensory neuron (OSN) class continuously express a single odorant receptor (OR) regardless of changes in the environment. However, little is known about the control of the robust, class-specific OR expression involved. Here, we investigate the cis-regulatory mechanisms and components that generate robust and OSN class-specific OR expression in *Drosophila*. Our results demonstrate that the spatial restriction of expression to a single OSN class is directed by clusters of transcription-factor DNA binding motifs. Our dissection of motif clusters of differing complexity demonstrates that structural components such as motif overlap and motif order integrate transcription factor combinations and chromatin status to form a spatially restricted pattern. We further demonstrate that changes in metabolism or temperature perturb the function of complex clusters. We show that the cooperative regulation between motifs around and within the cluster generates robust, class-specific OR expression.

## Author Summary

Our neurons can become over a hundred years old. Even if neurons are restructured and remodeled by their constant work of receiving, storing and sending information, they stay devoted to one single task and retain their identity for their whole life. How a neuron keeps its identity is not well understood. In the olfactory system, the identity of the olfactory sensory neuron (OSN) is a result of the expression of a single odorant receptor (OR) from a large receptor gene repertoire in the genome. Neurons that share an expressed receptor make a functional class. Here, we identify clusters of transcription factor binding motifs to be the smallest unit that drive expression in a single olfactory sensory neuron class. We further demonstrate that it is the structure of the cluster that determines the class specific expression. However, environmental stress, such as temperature changes or starvation, destabilizes the expression produced by the cluster. Our results demonstrate that stable expression is generated from redundant motifs outside the cluster and suggest that cooperative regulation generates robust expression of the genes that determine neuronal identity and function.

## Introduction

The expression of developmental genes is regulated such that they are either on or off at the appropriate time and in the correct place. The expression patterns of these genes must be robust; they must be stable and resistant to changes in both external and internal environments [1]. Mechanisms underlying developmental buffering and resistance to temperature changes and mutation have been described. Redundant enhancers act together to support gene expression and robustness under adverse conditions [2]. microRNAs silence ectopically expressed transcripts and buffer steady-state gene expression by controlling the levels of repressors or activators [3]. For genes expressed in mature cells, the demand for robust expression and spatial regulation is even more pronounced. Neurons, for example, are remarkably robust: their function can be maintained for one hundred years, implying that gene function is also maintained during this time. How the high requirement for stability is integrated into the continuous gene regulation that occurs in mature cells is poorly understood.

The high regulatory demands placed on the nervous system are typified by the olfactory sensory system, in which each olfactory sensory neuron (OSN) expresses only one olfactory receptor (OR) gene from its genomic repertoire of one hundred to one thousand ORs [4–6]. OSNs expressing the same OR project their axons to the same glomerulus in the brain and create a functional unit, the OSN class, that exists in both insects and mammals [6]. The restriction of OR expression to a single OSN class is crucial for the perception of odors as changes in OR expression pattern produce a mix of ORs in each OSN class, distorting the response properties of the class and thereby impairing odor detection [7].

Despite the difference in the number of ORs between mouse (1432 OR genes) and *Drosophila* (62 OR genes), there are common themes in the regulation of OR expression in these model organisms. In addition to class-specific expression, ORs exhibit spatially restricted expression patterns in each olfactory tissue [8,9]. OR gene expression is regulated by a small number of transcription factors (TFs) [10–17], and in *Drosophila*, these TFs regulate OR expression in a combinatorial fashion [16]. Directly upstream of each OR, there is a short cis-regulatory region sufficient for driving expression in OSNs but generally is insufficient for restricting expression to a single OSN class [16,18–20]. Searches for DNA binding motifs in OR cis-regulatory regions have not identified a direct regulatory “code” but, rather, an enrichment of motifs upstream of regulated OR genes [16]. Similarly, the identified TFs in *Drosophila* show little of the spatial and temporal specificity expected for combinatorial regulation, implying that it is neither the presence of a TF in an OSN nor the motif upstream of the OR that restricts expression to one OSN class.

Class-specific OR expression is generated in *Drosophila* in part by input from upstream repressive regions [16,20]. In vertebrates, a single OR allele is expressed in each OSN class [21,22]. The expression of a functional vertebrate OR creates a negative feedback loop [23–25] that reduces the expression of the H3K9 demethylase Lsd1 [26] and locks the expressed OR allele into a stable, robust expression state while suppressing the expression of other ORs [27]. Without monoallelic expression, vertebrate OR expression is not stable and robust within a single OSN class [18]. *Drosophila* ORs, like the majority of genes, are not expressed monoallelically and lack a feedback system, suggesting that other mechanisms must exist to ensure robust, specific biallelic gene expression.

Here, we address the cis-regulatory mechanisms that result in the precise and robust expression of *Drosophila* ORs. We utilize the short, well-defined cis-regulatory regions upstream of ORs that limit expression to a single class and the fact that projections from each OSN class form a stereotyped pattern, enabling the direct visualization of expression specificity. Our results demonstrate that structured motif clusters involving one to several TFs located directly upstream of the OR gene provide spatially restricted regulation to a single OSN class. We also show that cooperative gene regulation is a mechanism by which expression variability is buffered and the correct expression of ORs is ensured.

## Results

Previously, we showed that TFs bind to motifs upstream not only of ORs that they regulate but also of non-regulated ORs [16], suggesting that specific regulation requires a structure or order of motifs. A typical TF DNA binding motif is 8–10 bps in length and consists of a central 3–4 bp core motif flanked by a 2–4 bps segment with a supporting function [28,29]. To identify possible motif patterns, we determined the location of known core motifs in 32 different OR upstream regions. The scan included five core motifs bound by three TFs that regulate OR gene expression (S1 Table). All motifs were found upstream of all analyzed ORs, with the exception of the regulatory region of Or19a, which lacked E-boxes (the Fer1 motif).

### A cluster of Acj6^Hox/^Pou motif dimers regulates *Or85a* expression

**Fig 1:**
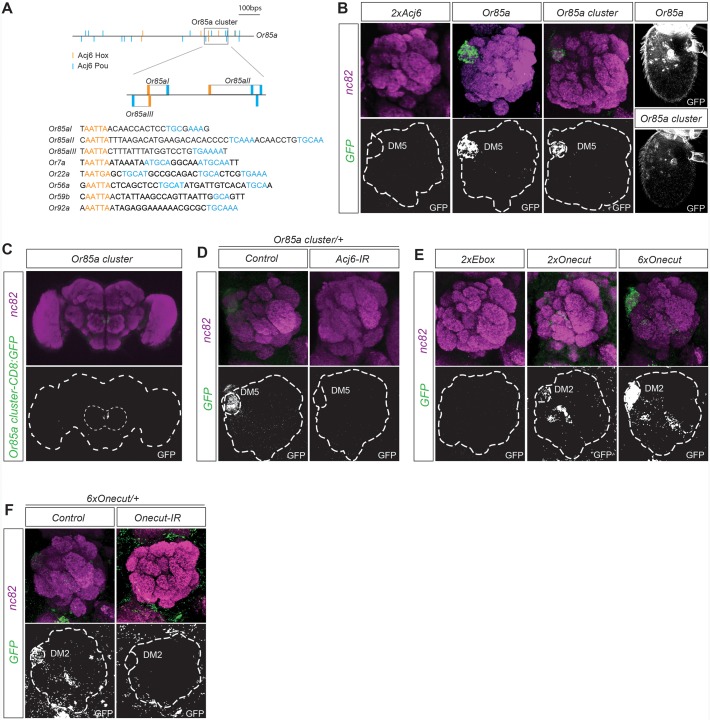
Motif clusters regulate OR class-specific expression. (A) Diagram depicting the 1000-bp upstream region of the *Or85a* gene and the position of the Pou (blue) and Acj6^Hox^ (orange) motifs in this region. The gray box indicates the 117-bp cluster of *Acj6* ^Hox^/pou motifs. Below is the alignment of the three *Or85a* Hox/pou clusters with examples of Acj6^Hox^/pou clusters from other OR regulatory regions. (B) Whole-mount brain staining shows the expression of GFP (green) driven by the *Or85a* cluster. Synaptic neuropil regions are labeled with the presynaptic marker nc82 (magenta). The marked region defines the whole brain and the antennal lobes. Below each merged image, the GFP channel is shown. (C) The GFP expression pattern driven by the *Or85a* cis-regulatory region and *Or85a* cluster in the antennal lobe and antenna. Note that synthetic clusters of tandem Acj6 binding motifs do not produce any GFP expression. (D) *Or85a* cluster-driven expression is lost in *Acj6-IR* (IR-inverted repeat). Control flies were crossed to *Peb-Gal4*. (E) GFP expression driven by synthetic clusters of tandem E-boxes and 2 or 6 Onecut Hox/cut motif dimers. Note the dose-dependent expression. (F) Loss of expression of the 6×Onecut cluster is observed in *Onecut-IR* flies. Control flies were crossed to *Peb-Gal4*.

To identify putative motif patterns that regulate OR expression, we focused on the regulation of the *Or85*a gene. The *Or85a* upstream region lacks published DNA binding motifs for Acj6, the sole TF regulating its expression. Acj6 and its vertebrate orthologs have two DNA binding domains, Hox and Pou, which bind two very different sequences: the Hox core motif (AATTA; [30–32]) and the Pou core motif (TGCAA/T; [29,33]), respectively. Within the first 1000 bp upstream of *Or85a*, we identified 17 Pou and 7 Hox core motifs. Several of the Hox and Pou motifs exhibited a possible dimer arrangement. A search of all 32 analyzed OR upstream regions showed an array of similar Hox/Pou dimers with variations in the spacing between the motifs (exemplified in Fig. 1A). Constructs with pairs of Acj6^Hox/^Pou dimers placed upstream of a synthetic minimal promoter fused to *CD8*:*GFP* did not induce expression (Figs. 1B and S1B), indicating that motifs from other TFs or spatial arrangements support Acj6 dimer function. Three of the Acj6^Hox^/Pou motif dimers upstream of *Or85a* generated a condensed cluster (Fig. 1A). To test whether the cluster was sufficient for expression in OSNs, we placed the cluster directly upstream of a minimal promoter fused to *CD8*:*GFP*. The *Or85a* Acj6 dimer cluster resulted in GFP expression specific to Ab2b OSN class neurons, which express *Or85a* and innervate DM5 (Fig. 1B, C). All insertions of the transgene resulted in equally strong and specific expression (S2 Table), demonstrating that the cluster is sufficient for expression in the correct OSN class, independent of the locus of insertion. Knockdown of *acj6* abolished the expression of the construct (Fig. 1D), showing that Acj6 likely binds the Hox/Pou dimers and the cluster then regulates the expression of *Or85a*.

### Onecut motif dimers produce OSN class-specific expression

To identify the smallest regulatory unit sufficient for OSN expression, we generated synthetic motif constructs. Constructs with pairs of E-boxes did not induce expression (Fig. 1E), indicating that E-boxes are insufficient for OR gene regulation. Onecut binds to Cut and Hox motifs spaced 2–6 bps apart [29]. A pattern scan revealed fixed Hox/Cut dimers upstream of 71% of the OR genes regulated by Onecut compared with 8% of those not regulated by Onecut, almost a tenfold enrichment. To investigate whether Hox/Cut dimers are sufficient to drive gene expression in OSNs with Onecut-regulated OR genes, we made constructs with two or six dimers directly upstream of a minimal promoter fused to *CD8*:*GFP*. The Onecut Hox/Cut dimers produced expression specific to Ab3a OSN class neurons, which express Or22a and innervate the DM5 glomerulus (Fig. 1E). Interestingly, Or22a expression is regulated by Onecut [16], and in our pattern scan, the closest Hox/Cut dimer to the consensus was found upstream of Or22a. Increasing the number of Hox/Cut dimers in the construct from 2 to 6 increased the expression level in Ab3a OSNs as well as the number of insertions that were expressed (Fig. 1E and S2 Table). Knockdown of *Onecut* attenuated the expression driven by the sextet (Fig. 1F), indicating a direct regulation by Onecut. Our results thus show that motif dimers specific to one TF provide sufficient regulatory information to specifically drive gene expression in a single OSN class.

### A motif cluster incorporates the combinatorial cis regulation of *Or59b*

**Fig 2:**
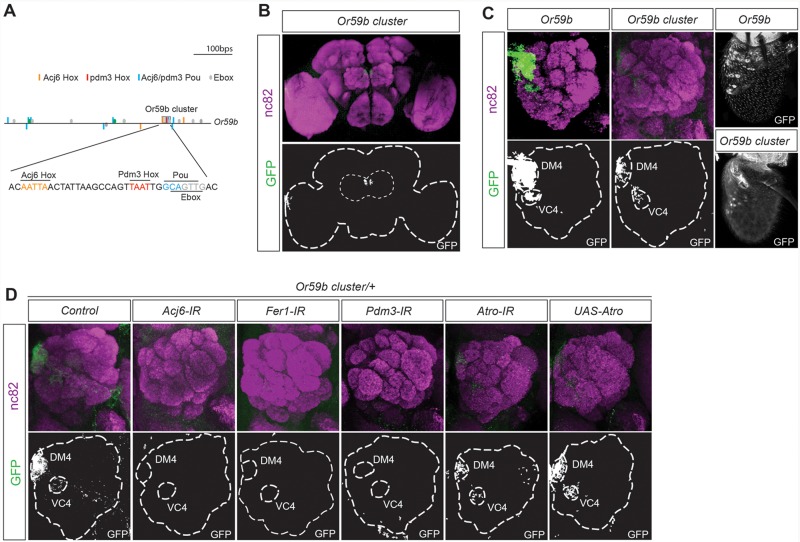
One motif cluster combines the TF regulation of *Or59b* gene expression. (A) Diagram of the 1000-bp upstream region of the *Or59b* gene showing the locations of the Pou (blue), Acj6^Hox^ (orange), Pdm3^Hox^ (red) and E-box (green) motifs. The gray box marks the cluster of Hox/pou/E-box motifs. Below, the 36-bp *Or59b* cluster sequence is presented. (B-D) A whole-mount brain shows GFP expression driven by the *Or59b* cluster (green) and the synaptic neuropil marked by nc82 (magenta). The marked region defines the whole brain and the antennal lobes. (C) GFP expression from the *Or59b* reporter and *Or59b* cluster in the antenna and in the antennal lobe, where it marks axonal projections to the DM4 and VC4 glomeruli. (D) Loss of expression produced by the *Or59b* cluster is observed in the *Acj6-*, *Fer1-* and *Pdm3-IRs* but not in *Atro-IR* or *UAS-Atro* overexpression lines. Control flies were crossed to *Peb-Gal4*.

To identify how the combinatorial input stemming from multiple TFs regulates OR gene expression, we focused on *Or59b*, whose expression is regulated by three factors with known DNA binding properties: *acj6*, *Fer1* and *pdm3* [16] (S2 Fig.). Pdm3 binds to a Hox motif (TAAT) 2–3 bp upstream of a Pou motif (TGCAA/T) [34]. One Pdm3 Hox/Pou motif dimer was identified upstream of *Or59b*. Interestingly, the Pdm3 Pou motif overlapped with an E-box, the motif that binds bHLH proteins such as Fer1, and was directly downstream of one of the two Acj6^Hox^ motifs (Fig. 2A), indicating that the motifs for all three TFs that regulate *Or59b* are clustered. This small, 36 bp cluster drove expression in between 30 and 50 OSNs in the proximal region of the antenna (Fig. 2C). Analysis of the axonal projections to the antennal lobes showed that the *Or59b* cluster produced expression was confined to two OSN classes: Ab2a, which expresses *Or59b* and innervates the DM4 glomerulus, and Ab7b, which expresses Or67c and innervates the VC4 glomerulus (Fig. 2B, C). Knockdown of the 3 TFs that regulate Or59b expression, *acj6*, *Fer1* and *pdm3* resulted in the loss of *Or59b* cluster produced expression in both OSN classes (Fig. 2D), implying that this TF combination does not segregate the Ab2a and Ab7a classes. We have previously shown that the co-repressor Atro represses *Or59b* expression specifically in the Ab7a OSN class [35]. Overexpression or knockdown of *Atro* did not attenuate reporter expression driven by the *Or59b* cluster in the Ab7a OSN class (Fig. 2D), demonstrating that Atro represses *Or59b* expression via a mechanism that is separate from the cluster and that restricts OR expression to a single class.

### The ratio of Acj6 and Pdm3 specifies the *Or59b* cluster-produced expression

**Fig 3:**
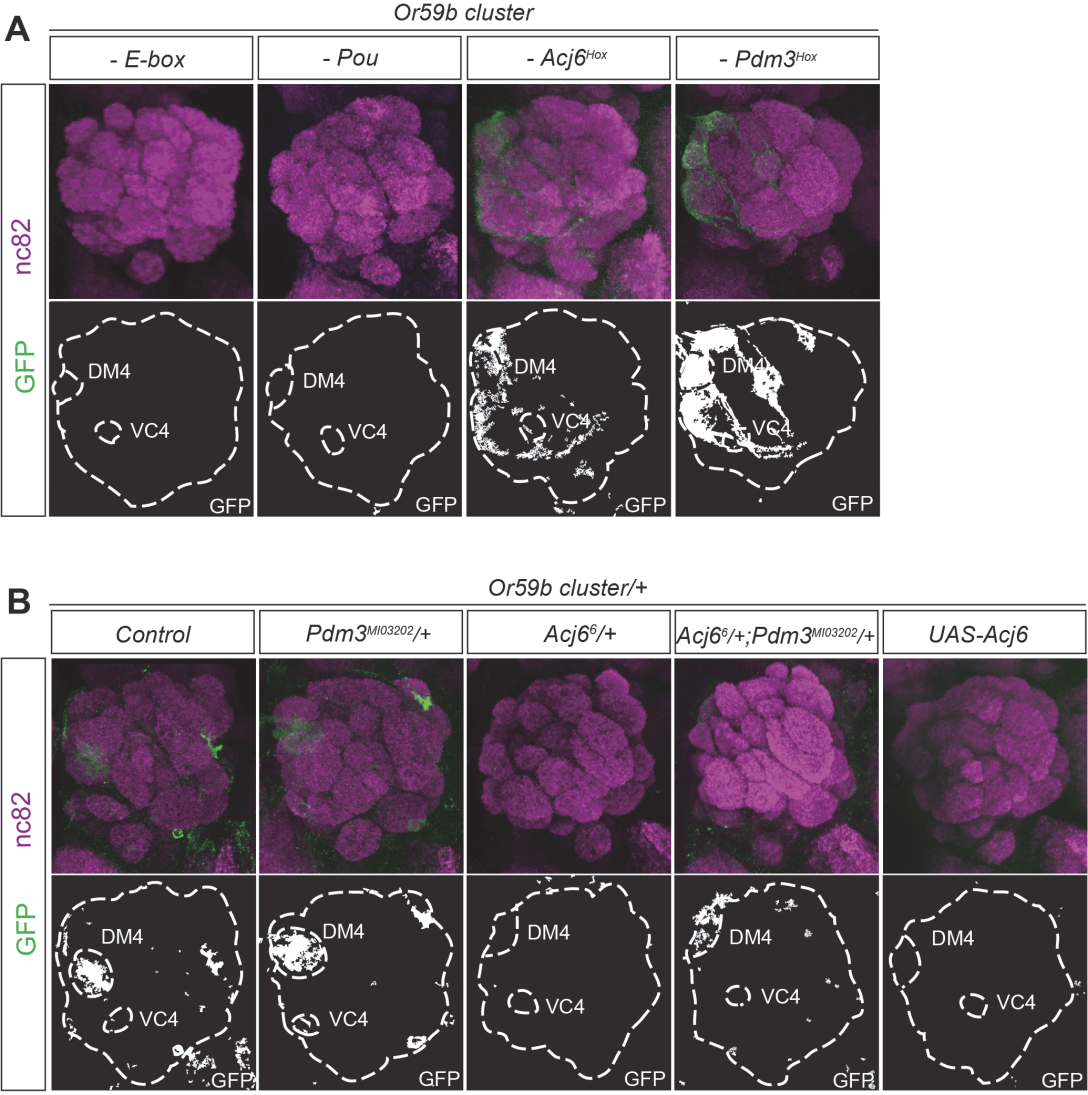
The Acj6 to Pdm3 ratio dictates the *Or59b* cluster-driven expression. (A) GFP (green) expression driven by *Or59b* cluster constructs with mutated Acj6^Hox^, Pdm3^Hox^, Pou and E-box motifs. Synaptic neuropil regions are labeled with the presynaptic marker nc82 (magenta). (B) GFP expression driven by the *Or59b* cluster in different backgrounds. Loss of expression of the *Or59b* cluster is observed in *acj6* ^*6*^ +/− flies and is rescued in *acj6^6^/pdm3^MIO3202^* flies. Control flies were crossed to *w* ^*1118*^. Schematic interpretations of the results are presented in S5 Fig.

To investigate the regulatory function of each TF, we made constructs with mutations in the different motifs belonging to the *Or59b* cluster. Mutation of the E-box resulted in a total loss of reporter expression (Figs. 3A and S3A), indicating that bHLH proteins induce its expression. Mutation of the Acj6 and Pdm3 Pou motif caused loss of expression, whereas mutation of each Hox motif produced ectopic expression (Figs. 3A and S3A), indicating that the repressive or inductive function of Acj6 and Pdm3 is dictated by the Hox motif. Further genetic analyses demonstrated that *pdm3* is downstream of *acj6* and that both can either repress or activate the cluster function and expression (S3B–S3C Fig.). To explore whether the cluster interprets the protein levels of Pdm3 and Acj6, we manipulated the level of each factor. The expression of the cluster was sensitive to the loss of one copy of *acj6* but not of *pdm3* (Fig. 3B). The loss of expression was rescued by lowering the copy number of both factors (Fig. 3B), demonstrating that the ratio of Acj6 to Pdm3 creates a window of cluster function that limits *Or59b* expression to two OSN classes.

### The structure of the *Or59b* cluster generates OSN class-specific expression

**Fig 4:**
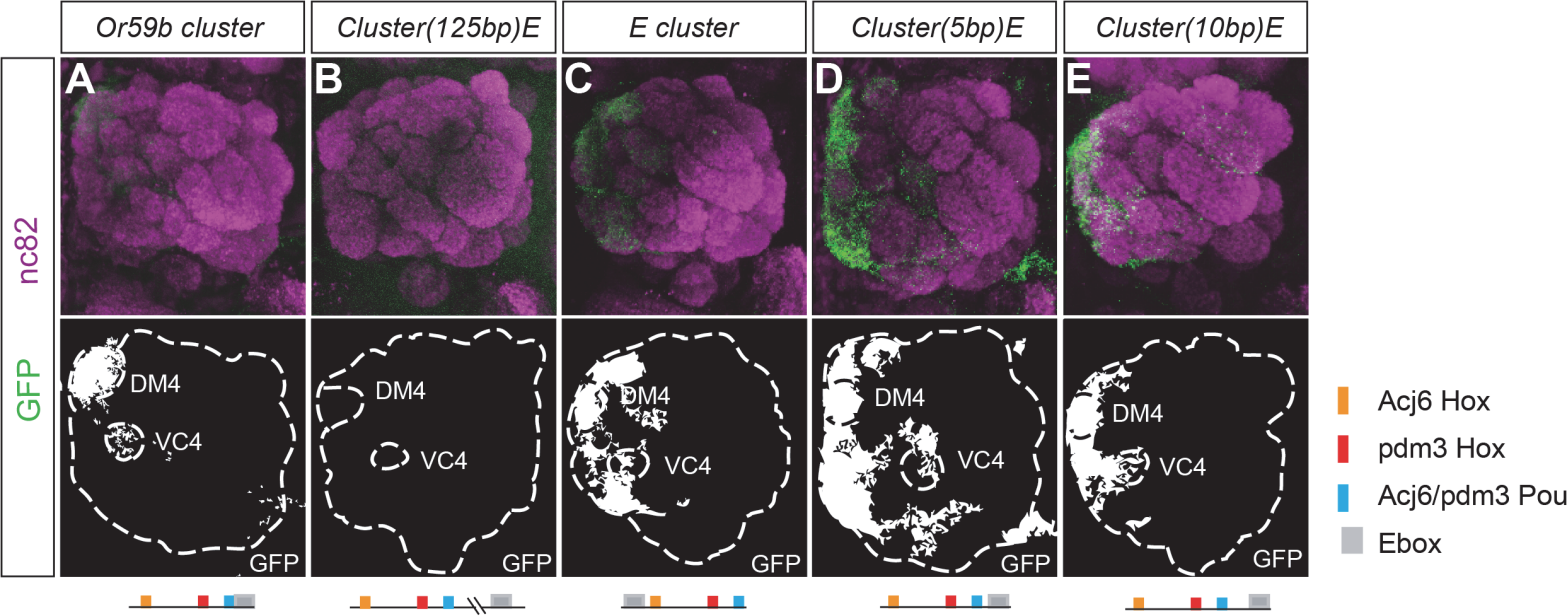
Spatial expression pattern is dictated by the structure of the *Or59b* cluster. GFP expression (green) produced by (A) the *Or59b* cluster, (B) the cluster with the E-box displaced 125bps, (C) with the E-box 10bps upstream the cluster, (D-E) the Ebox 5 or 10 bp downstream the cluster. Synaptic neuropil regions are labeled with the presynaptic marker nc82 (magenta). A schematic representation of different rearrangements is shown under each figure. Schematic interpretations of the results are presented in S5 Fig.

To investigate whether cluster structure regulates expression, we rearranged the order of the motifs in the *Or59b* cluster. First, we moved the E-box 125 bp downstream the cluster, which disrupted expression (Figs. 4B and S4A), demonstrating that the combinatorial clustering of the motifs was required for expression. Next, we addressed the regulatory function of the overlap between the Pou motif and the E-box. Moving the E-box upstream of the cluster caused ectopic expression in seven OSN classes, Ab1a, Ab2a, Ab3a, Ab5b, Ab7b, Ab8a, Ab8b and Ab10b (Figs. 4C and S4A), indicating that the precise location of the E-box dictates a repressive function necessary for class-specific OR expression. To further address how TFs binding at the E-box and Pou motifs interact, we moved the E-box either one-half or a full DNA turn (5 or 10 bp, respectively, S1C Fig.) that placed the TFs at different phases and sides of the DNA. Both constructs resulted in stereotyped ectopic expression in the seven OSN classes (Figs. 4D, E and S4A). As both the Ebox and the Pou motifs were shown to be required for cluster function (Fig. 3A), the above results indicate that occupancy of either of the two motifs interferes with the other and causes the repression of expression. Further genetic analyses placed Fer1 downstream of the Hox/Pou factors (S4B–S4C Fig.). Together, these results demonstrate that the composition and relative positions of motifs within the cluster define and restrict the expression of *Or59b*.

### Starvation and low temperature destabilize *Or59b* cluster function

**Fig 5:**
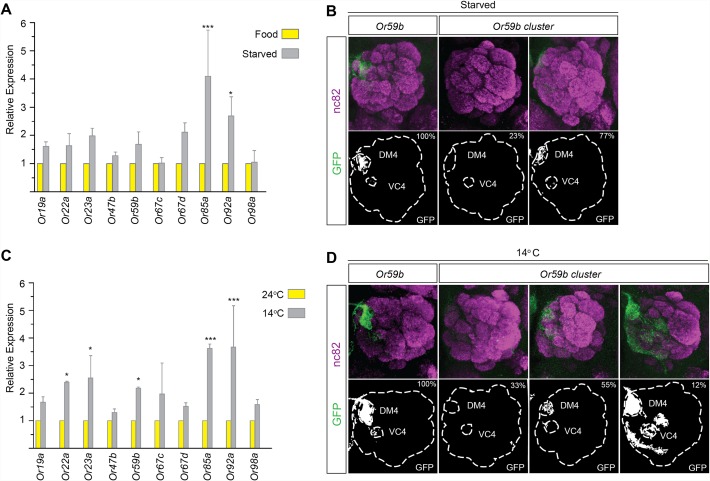
Temperature stress or starvation hampers *Or59b* cluster function. (A) Following 3 days of starvation, the mRNA levels of indicated OR genes were measured by qPCR and compared with control flies (* p < 0.05; ** p < 0.01; error bars represent SEM). (B) GFP expression (green) driven by the *Or59b* reporter or *Or59b* cluster following 3 days of starvation. Synaptic neuropil regions are labeled with the presynaptic marker nc82 (magenta). Phenotype penetrance is marked as a percentage at the top-right corner of the image. (C) Following 3 days at 14°C, the mRNA levels of the indicated ORs were measured by qPCR and compared with flies maintained at 24°C (* p < 0.05; ** p < 0.01; *** p < 0.001; error bars represent SEM). (D) GFP expression produced by the *Or59b* reporter and the *Or59b* cluster at 14°C. Note that the same insertion of the *Or59b* cluster construct produces both loss- and gain-of-expression phenotypes at 14°C. Schematic interpretations of the results are presented in S6 Fig.

For proper odorant perception, OR expression must be active continuously and must be restricted to a single OSN class, despite changes in the environment. To investigate OR gene expression and class-specific transcription under conditions of environmental fluctuation, we first starved flies for three days. qPCR revealed that the mRNA levels of most ORs increased slightly upon starvation (Fig. 5A). Starvation did not change the expression produced by reporter transgenes with the cis regulatory region between *Or59b* and the gene upstream fused to *CD8*:*GFP* (Fig. 5B and S3 Table), showing that robust class-specific expression is encoded by the region directly upstream the gene. Interestingly, starvation attenuated the expression of the *Or59b* cluster (Fig. 5B and S3 Table). These results show that the cluster lacks the regulatory information required to maintain class-specific expression during starvation.

To further investigate the requirements for robust class-specific OR expression, we changed the physical environment of the flies. Flies are stressed by high (>30°C) and low (<15°C) temperatures [36]. We switched flies between 3 and 5 days old to low temperature (14°C) and kept a control group at ambient temperature (24°C) for 3 days. qPCR showed increased mRNA levels of most assayed ORs in flies exposed to low temperature compared with those kept at ambient temperature (Fig. 5C). The *Or59b* cis-regulatory region drove reporter expression at both low and ambient temperatures (Fig. 5D). Different cluster insertions produced similar expression phenotypes, with stable expression at the ambient temperature, but at low temperature, the cluster produced ectopic expression in several OSN classes in 12%, no expression in 33% and restricted class specific expression in 55% of the analyzed animals (Fig. 5D and S3 Table). These results show that the cluster can support class-specific expression under ambient conditions, but the fine-tuned balance of TF assembly is perturbed in low-temperature or starvation conditions.

### Cooperative gene regulation generates robust class-specific OR expression

**Fig 6:**
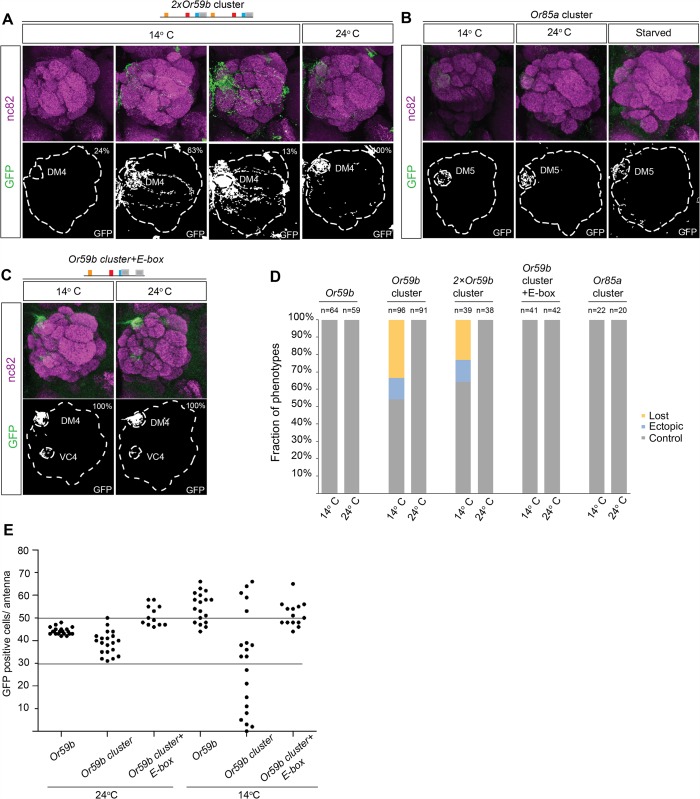
Cooperative regulation between the cluster and surrounding motifs produces robust class-specific expression. (A) GFP expression (green) driven by the *2×Or59b* cluster at 14°C and 24°C. Note the three expression phenotypes produced by the *2×Or59b* cluster at 14°C. Synaptic neuropil regions are labeled with the presynaptic marker nc82 (magenta). (B) GFP expression (green) produced by the *Or85a* cluster at 14°C, 24°C or following 3 days of starvation. Note that GFP expression is equally strong in different lines at 14°C. (C) The *Or59b* cluster with an additional E-box produces robust expression at 14°C and 24°C. (D) The fractions of the brains showing stable or bimodal expression of GFP according to the genotype and temperature. Note that only the *Or59b* cluster and 2×*Or59b* cluster are unstable at 14°C. (E) Quantification of GFP positive cells in the antenna. *Or59b* cluster shows a varied number of GFP positive cells at 14°C compared to 24°C. *Or59b* and *Or59b* cluster with an additional E-box show stable expression in both temperatures. Schematic interpretations of the results are presented in S6 Fig.

Because the *Or59b* cluster produces weak expression compared with the Or59b reporter (Fig. 2C), we investigated whether the level of *Or59b* expression generates robust class-specific expression. Two tandem copies of the *Or59b* cluster produced strong expression at ambient temperature and both loss and gain of expression phenotypes at low temperature (Fig. 6A, D), demonstrating that expression level does not buffer against environmentally induced changes and that class specificity is maintained via a separate mechanism. Interestingly, environmental changes did not affect the function of the *Or85a* cluster (Fig. 6B, D), suggesting that cooperative binding of one TF may be sufficient to drive robust class-specific expression. Of the three TFs known to regulate Or59b, Fer1 has 10 binding sites (E-boxes) outside the cluster that can cooperate with the cluster to regulate *Or59b* expression (Fig. 2A). To test whether cooperating E-boxes might produce robust *Or59b* cluster expression, we generated a cluster with two E-boxes. The addition of an extra E-box led to stabilized expression in flies challenged with changes in temperature or food (Fig. 6C, D). A count of GFP-positive OSNs showed that the cluster produced a varied number, from a few cells to over 60 positive cells per antenna, at 14°C (Fig. 6E). The number of positive cells per antenna was fully rescued to the control number by the addition of an extra E-box, demonstrating that the cooperative function of single motifs around a cluster can stabilize the assembly and expression produced by the cluster.

### Epigenetic state controls *Or59b* cluster function

**Fig 7:**
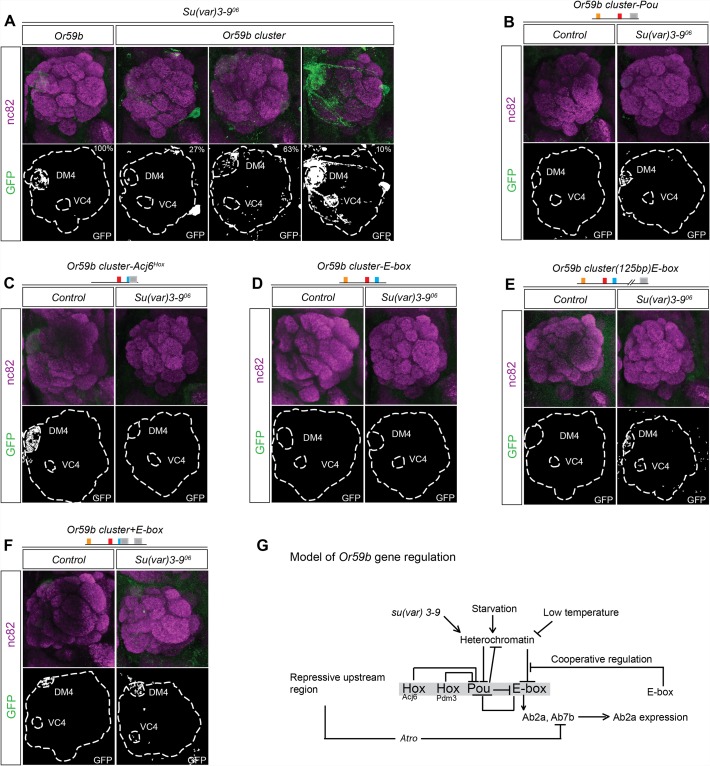
Heterochromatin modulation of the *Or59b* cluster. (A) GFP expression (green) driven by the *Or59b* reporter or *Or59b* cluster in *su(var)3–9* ^*06*^ heterozygote flies. Synaptic neuropil regions are labeled with the presynaptic marker nc82 (magenta). Control flies were crossed to *w* ^*1118*^. (B-E) GFP expression (green) driven by mutated *Or59b* cluster versions in *su(var)3–9* ^*06*^ heterozygote flies. Note that the loss of GFP expression driven by the *Or59b* cluster with a mutated Pou motif or with a distant E-box is rescued in *su(var)3–9* ^*06*^ heterozygote flies. (F) GFP expression (green) driven by an *Or59b* cluster with an additional E-box in *su(var)3–9* ^*06*^ heterozygote flies. Note that the E-box rescues the produced *su(var)3–9* ^*06*^ expression phenotypes. (G) Model depicting the function of the cluster in the regulation of *Or59b* expression. Our results propose that the Hox/Pou motif regulates the heterochromatin state and allows bHLH proteins to bind the E-box, which induces expression. The E-box and Pou motif sequences overlap to generate unstable binding, and a steady state is generated that drive expression in the Ab2a and Ab7b OSN classes. Cooperative interactions between E-boxes stabilize expression in the face of environmental perturbations. Schematic models of the results are presented in S5–S6 Figs.

The variability of expression produced by different *Or59b* cluster insertions (S2 Table) and the general increase of OR expression upon environmental changes suggested a general epigenetic mechanism for regulation. In mice, H3K9 trimethylation, a marker of heterochromatin, is required for stable and robust class-specific OR expression [27,37]. To address whether changes in H3K9 trimethylation control *Or59b* cluster function, we introduced a mutant allele of *su(var)3–9*, the enzyme that trimethylates H3K9 [38]. *Or59b* reporters showed robust expression in *su(var)3–9* heterozygote flies (Fig. 7A). By contrast, each *Or59b* cluster insertion showed both gain- and loss-of-expression phenotypes in *su(var)3–9* ^*+/−*^ flies (Fig. 7A), indicating that H3K9 methylation status modulates gene expression driven by the cluster.

To address how TF assembly at the cluster interacts with the assembly of heterochromatin, we crossed the various cluster versions to the *su(var) 3–9* mutant. In the heterozygote *su(var)3–9* background the ectopic expression of the Acj6^Hox^ mutant cluster was lost (Fig. 7C), indicating that the epigenetic status at the cluster determines the function of Acj6 and Pdm3. Moreover, the attenuated expression of the Pou mutant cluster was weakly rescued in the heterozygote background (Fig. 7B), indicating that the Hox/Pou TFs generate the open chromatin required for the induction of *Or59b* expression. Interestingly, the loss of expression by the mutated E-box was not rescued in *su(var)3–9* heterozygotes (Fig. 7D), placing the heterochromatin regulation downstream of the Hox/Pou factors and upstream of the E-box. The loss of heterochromatin in the heterozygote background further induced expression of the cluster with the E-box displaced by 125 bp (Fig. 7E), supporting the notion that Hox/Pou factors open chromatin and allow for the binding of bHLH proteins to the E-box (modeled in Figs. 7G and S5). As with the temperature- and starvation-induced phenotypes, the additional E-box construct rescued the *su(var) 3–9* phenotypes (Fig. 7F), implying that stabilization of TF binding at the E-boxes buffers the cluster function. Our results thus support a model in which the Hox- and Pou-binding proteins open chromatin and let bHLH proteins to bind the E-box that induce expression. The bHLH binding compete with the Hox/Pou proteins and cooperation between additional E-boxes stabilize bHLH binding and buffers *Or59b* expression from variation in epigenetic and environmental states (Fig. 7G; regulatory models of each phenotype are presented in S5–S6 Figs).

## Discussion

Here, we demonstrate that clusters of low-affinity motifs generate restricted OR expression. We show that the composition of the motif cluster sets requirements for expression that are only fulfilled by the OSNs of one or a few classes. We further show that TFs with two DNA-binding domains can recognize simple motif clusters that restrict expression to a single class and that complex clusters with motifs corresponding to multiple TFs integrate the combinatorial information from TFs and chromatin status to produce class-restricted expression. Finally, we show that cooperation among redundant motifs, or perhaps even clusters, generates the robust single class OR expression required to support odorant detection throughout the life of the organism.

### Low-affinity motifs can be advantageous for gene regulation

The motifs identified in this study are short, are likely to have low affinity and are abundant in OR cis-regulatory regions. Nonetheless, these motifs are sufficient to regulate the restriction of OR expression to a single class. Our results further imply that low information value of a motif can be an advantage for several reasons. First, TF binding to low-affinity motifs requires cooperative input for stability, thus favoring combinatorial and patterned gene regulation. Second, a high on/off rate supports competition among TFs at overlapping motifs, which we show is crucial for the integration of combinatorial input from several TFs to restrict OR transcriptional output to a single class. Third, weak TF interactions with the motif facilitate direct chromatin regulation of the locus [39]. Finally, the level of degeneracy of each motif defines the role, and possibly the function, of the TF in each cluster, which increases the use and flexibility of a TF from a static activator to a modulator, as we showed for Acj6/pdm3 by demonstrating both repressive and inductive roles.

Short motifs are evolutionarily unstable because they can be generated or lost with one or two mutations. The olfactory system is evolutionarily very plastic, with the continuous generation and loss of OR genes, implying that selection might regulate the birth and death of weak motifs. Even a complex cluster such as the *Or59b* cluster can only be found as a unit in the *melanogaster* clade and shows a large number of changes among species in the clade. Recently, it has been shown that the evolutionary stability of expression patterns differs between vital developmental genes and genes expressed only in mature cells [40,41]. Thus, one possibility is that short motifs are the product of an active selection process. Studies on the conservation of motifs upstream of genes expressed in different tissues and stages indicate that upstream genes expressed in adult tissues, such as the vertebrate liver [42,43], are less conserved than those critical for developmental processes, such as invertebrate segmentation [44,45], and organelle function, such as the Rfx regulation of genes conserved in cilia function [46]. It will therefore be interesting to determine whether the predictive use of conserved large (>8 bps) motifs is limited to the prediction of evolutionarily stable systems, such as development or organelle function, and whether the identification of motif patterns or clusters of core motifs will improve predictions regarding the regulatory function of non-vital genes expressed in mature cells.

### Regulation of spatial OR expression by motif clusters

Cooperative regulation of clustered motifs has been shown in *Drosophila* to restrict regulation by broadly expressed TFs to regions of the embryo [47–49]. Our results show that the simplest switches are motif clusters recognized by one TF. Tandem synthetic consensus Acj6 motif dimers did not result in any expression, but the more complex *Or85a* Acj6 cluster did, indicating that the structure of the motif cluster can limit the function of a broadly expressed TF to regulate OR gene expression to a single OSN class. Interestingly, despite the fact that *onecut* is expressed in several OSN classes [16], the cluster of Hox/cut motifs produced expression restricted to a certain class. As TFs with more than one DNA binding domain, such as Acj6 and Onecut (with two binding domains each), can bind to multiple DNA motifs simultaneously [29,30], the arrangement of motifs within a cluster can define expression pattern, and in this manner, a broadly expressed TF can produce a very restricted expression pattern.

The integration of several TFs requires more complex clusters. The *Or59b* cluster integrates the function of 3 TFs, Acj6 and Pdm3, which bind to two different Hox motifs and compete for one Pou motif, and Fer1, which binds to an E-box that partly overlaps with the Pou motif. Our results show that the competition between Hox/Pou proteins and bHLH proteins allows the cluster to integrate the epigenetic status and the levels of Hox/Pou proteins, which can be summarized in the following model: the Hox/Pou TFs Acj6 and Pdm3 compete for the Pou motif, where Pdm3 likely opens chromatin and facilitates the binding of bHLH proteins to the E-box, which induces *Or59b* expression. As the bHLH proteins bind the E-box, binding of the Hox/Pou TFs to the cluster is destabilized, reducing the opening of chromatin. In this less-favorable chromatin environment, binding to the E-box is reduced and a steady state is generated. The generated steady state is sufficient to support expression in two OSN classes; the Atrophin complex represses expression in one of two classes, resulting in expression of the *Or59b* gene in a single OSN class.

### Cooperative TF function generates robust class-specific OR expression

Various environmental challenges generated a stereotyped expression phenotype, indicating that a general molecular mechanism underlies robust OSN class expression. One cause of combined gain and loss of expression phenotypes is direct competition between a repressor and activator for one motif [50]. Our results demonstrate that it is the competition between the Hox/Pou TFs and bHLH proteins that generates unstable expression and that cooperative regulation of bHLH proteins bound to the E-box in the cluster and secondary E-boxes beyond the cluster stabilizes OR expression. This stabilization is likely a function of favoring bHLH binding to the E-box in the cluster, thereby reducing the need for Hox/Pou regulation and chromatin opening at the locus.

Even in simple clusters with only one TF motif, cooperative function generates robust expression in the class. Interestingly, robustness to environmental changes during development has been shown to be produced by shadow enhancers [2] (redundant cis-regulatory regions that together support expression [51]) or homotypic motif clusters [52], suggesting that cooperative regulation between motifs and clusters might be a general mechanism through which to maintain restricted gene expression.

## Materials and Methods

### Drosophila stocks

The *Pebbled-Gal4* (*Peb-Gal4*) and *acj6* ^*6*^ mutants were kind gifts from Liqun Luo (Stanford University, Stanford, CA, USA). The *su(var)3–9* ^*06*^ mutant was a kind gift from Anita Öst (Linköping University, Linköping, Sweden). The following fly lines were obtained from the Vienna Drosophila Center (VDRC; Vienna, Austria; http://stockcenter.vdrc.at): *Acj6-IR*, *Atro-IR*, *Fer1-IR*, *UAS-Atro*, and *UAS-Dcr2*. The following RNAi lines were obtained from the Transgenic RNAi Project (TRiP; Harvard Medical School, Boston, MA, USA; http://www.flyrnai.org): *Fer1-IR* (27737; 50672), *Onecut-IR* (29343), *Pdm3-IR* (35726, 26749). The following fly lines were provided by the Bloomington Drosophila Stock Center (BDSC; Indiana University, Bloomington, IN, USA; http://flystocks.bio.indiana.edu): *w* ^*1118*^, *UAS-tub-Gal80ts*, *Pdm3* ^*MI03202*^ (37337), *Pdm3* ^*MI01072*^ (37552).

### Bioinformatics

An online pattern search tool (http://www.bioinformatics.org/sms2/dna_pattern.html) was used to scan 1 kb upstream from the translational start site of each OR for 6 motifs recognized by 4 TFs: Acj6 (Hox and Pou), Fer1 (E-box), Onecut (cut and Pou) and Pdm3 (Hox linked to Pou, only for *Or59b*) (S1 Table).

### Cloning

All constructs were synthesized at Genescript and cloned into a transformation vector containing a synthetic TATA region fused to a single ORF that contained the mCD8 transmembrane domain, four tandem copies of GFP, and two c-*myc* epitope tags, as previously described (Couto et al). The DNA constructs were injected into *w* ^*1118*^ flies at BestGene, and six to 12 lines were analyzed per construct.

### RNAi methodology and environmental experiments

Virgin flies of the RNAi line were mated with males containing *Pebbled-GAL4*, *UAS-Dicer2*, and the cluster transgenes. The crosses were set up and maintained at 24°C and 2–5 d after eclosure, flies were dissected, stained and scored for phenotypes. RNA interference lines for *Acj6*, *Atro*, *Fer1*, and *Onecut* were previously described [16,35]. Both *pdm3* RNA interference lines produced identical phenotypes that were phenocopied by the Pdm3 mutant (*Pdm3* ^*MI01072*^).

All flies were raised on standard Drosophila culture medium at 24°C and collected 2–5 days after eclosion unless otherwise specified. *w* ^*1118*^ flies were used as controls. In the experimental group, flies were transferred to new vials and maintained for 3 days at 14°C. In the starvation experiments, 2–5-day-old flies were kept in a vial with water-soaked filter paper for 3 days.

### Immunofluorescence

Immunofluorescence was performed according to previously described methods [16]). The following primary antibodies were used: rabbit anti-GFP (1:2000, TP-401; Torrey Pines Biolabs) and mouse anti-nc82 (1:100; DSHB). Secondary antibodies were conjugated with Alexa Fluor 488 (1:500; Molecular Probes). Confocal microscopy images were collected on an LSM 700 (Zeiss) and analyzed using an LSM Image Browser. The numbers of co-expressing BP104 and GFP OSNs for different constructs were counted from the images. Adobe Photoshop CS4 (Adobe Systems) was used for image processing.

### qPCR

Antennae were obtained with a sieve after freezing 2–5-day-old flies in liquid nitrogen. Total RNA from antennae was extracted with TRIzol reagent (Invitrogen) followed by purification with the RNeasy kit (Qiagen). Quantitative PCR was conducted on an Applied Biosystems 7900HT real-time PCR system (Life Technologies) using the Power SYBR Green PCR master mix (Applied Biosystems, Life Technologies) and primer sets designed using Primer Express software v3.0.1 (Integrated DNA Technologies). Tubulin was used as an internal control for the experiments. To amplify cDNA products and not genomic DNA, primers were designed to join the end of one exon with the beginning of the next exon. Quantitative PCR for each primer set was performed on both control and experimental samples for 40 cycles. Following amplification, melt curve analysis and ethidium bromide agarose gel electrophoresis were performed to evaluate the PCR products. The relative quantification of the fold change in mRNA expression was calculated using the 2−ΔΔCT threshold cycle method.

## Supporting Information

S1 Fig Clusters and construct sequences. Acj6^Hox^ motifs in orange, Pdm3^Hox^ motifs in red, Pou motifs in blue, the E-box motifs in green and Onecut Hox/Cut motifs in purple. (A) *Or85a* cluster sequence (B) synthetic motif sequences (C) *Or59b* cluster versions, substitutions or deletions are marked in gray.
(TIF)

S2 Fig Pdm3 regulates *Or59b* expression. In *Pdm3*-knockdown flies, expression of the *Or59b* reporter is lost (GFP, green). Synaptic neuropil regions are labeled with the presynaptic marker nc82 (magenta).
(TIF)

S3 Fig Epistasis experiments of Acj6 and Pdm3 regulation of the *Or59b* cluster. (A) Antennae that show the GFP expression produced by *Or59b* clusters with different mutations. (B) GFP expression (green) produced by an *Acj6* ^*Ho*x^ motif mutated cluster in different genetic backgrounds shows that the mutation made the cluster independent of Acj6 and placed *pdm3* genetically downstream of *acj6*, as produced expression was lost in the *Pdm3-IR* flies. (C) GFP expression (green) produced by a Pdm3^Hox^ motif-mutated cluster in different genetic backgrounds. The loss of ectopic expression in *Pdm3* mutant heterozygote flies revealed that the auxiliary function of *Acj6* is sufficient to support expression in the Ab2a and Ab7b classes. Overexpression of *Acj6* attenuated the ectopic expression of the *Or59b* cluster, supporting a repressive function for both Acj6 and Pdm3. Synaptic neuropil regions are labeled with the presynaptic marker nc82 (magenta) in both B and C.
(TIF)

S4 Fig Genetic analysis of the Or59b cluster regulation by Fer1 and the E-box motif. (A) Antennae that show the GFP expression produced by *Or59b* clusters with a shifted or dislocated E-box. (B) GFP expression (green) produced by an *Acj6* ^*Ho*x^ motif mutated cluster. The partial but not full loss of expression in the *Fer1-IR* background indicates a weak redundant bHLH regulation repressed by Acj6. (C) GFP expression (green) produced by a Pdm3^Hox^ motif mutated cluster. The total loss of ectopic GFP expression in the *Fer1-IR* background indicates that a combination of Pdm3 and Fer1 drives expression in the ectopic OSN classes and places Fer1 downstream of both Acj6 and Pdm3.
(TIF)

S5 Fig A model of *Or59b* cluster function and how the results of Figs. 3 and 4 can be predicted. (A) A model that summarizes the regulation events within the *Or59b* cluster. In short, the results show that Acj6 and Pdm3 bind two different Hox motifs and compete for a common Pou motif. We further show that the Hox interactions are repressive and that the Pou interaction is required to lift the suppression of the heterochromatin. We show that the E-box is downstream of Acj6, Pdm3 and the heterochromatin regulation. Our results further show that Pdm3 and Acj6 counteract the heterochromatin and likely allow binding to the E-box by bHLH proteins that in turn destabilize the POU interactions of Acj6 and Pdm3, establishing a steady state that supports expression in the Ab2a and Ab7b classes. (B-G) The models shown depict the predicted regulatory outcome of each genotype. Line thickness and blackness mark level of input (black, high input; light gray, low input).
(TIF)

S6 Fig Models of the *Or59b* cluster modulation of environmental perturbations or *Su(var)3–9* heterozygosity. (A-C) The models shown depict the predicted regulatory outcomes of each expression phenotype caused by starvation (A), temperature (B) and *su(var)3–9* ^*06*^ (reduced heterochromatin) (C). (D) The models shown depict the predicted interactions between the different mutant versions of the cluster and *su(var)3–9* ^*06*^. Reduced heterochromatin can generate increased binding at the Pou and E-box motifs. Rescue of the expression in the Pou mutant places the heterochromatin between the Pou and E-box and the output of the cluster downstream of the E-box. (E and F) The extra E-box stabilizes the expression of the *Or59b* cluster in *su(var)3–9* heterozygous flies and in flies at low temperature, indicating that cooperative regulation supports specific OR expression. Line thickness and blackness indicate levels of input (black, high input; light gray, low input).
(TIF)

S1 Table Core motifs used in the bioinformatics analysis.
(DOCX)

S2 Table List of transgenic flies lines.
(DOCX)

S3 Table Summary of the environmental phenotypes. The table presents the fraction of the flies with mentioned phenotype in various metabolic and environmental conditions.
(DOCX)

S4 Table Primer sequences for the qPCR.
(DOCX)
