# Supplementary figures and images for: Cis-Regulatory Mechanisms for Robust Olfactory Sensory Neuron Class-restricted Odorant Receptor Gene Expression in Drosophila
Source: PLoS Genet. 2015 Mar 11;11(3):e1005051. doi: 10.1371/journal.pgen.1005051 (PMC4356613; doi:10.1371/journal.pgen.1005051)

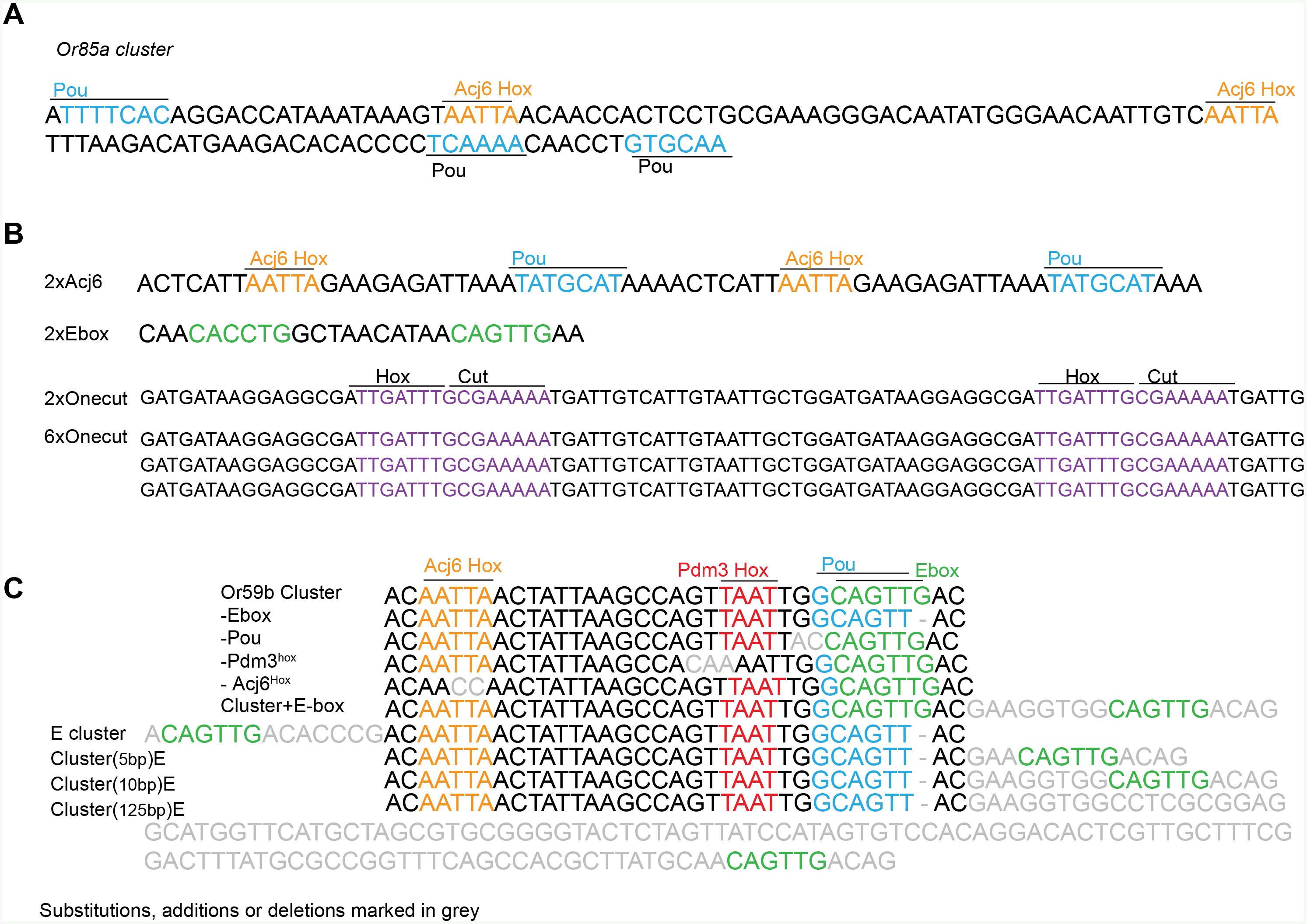

Supplement: S1 Fig — Clusters and construct sequences. Acj6Hox motifs in orange, Pdm3Hox motifs in red, Pou motifs in blue, the E-box motifs in green and Onecut Hox/Cut motifs in purple. (A) Or85a cluster sequence (B) synthetic motif sequences (C) Or59b cluster versions, substitutions or deletions are marked in gray. (TIF) [file pgen.1005051.s001.tif]

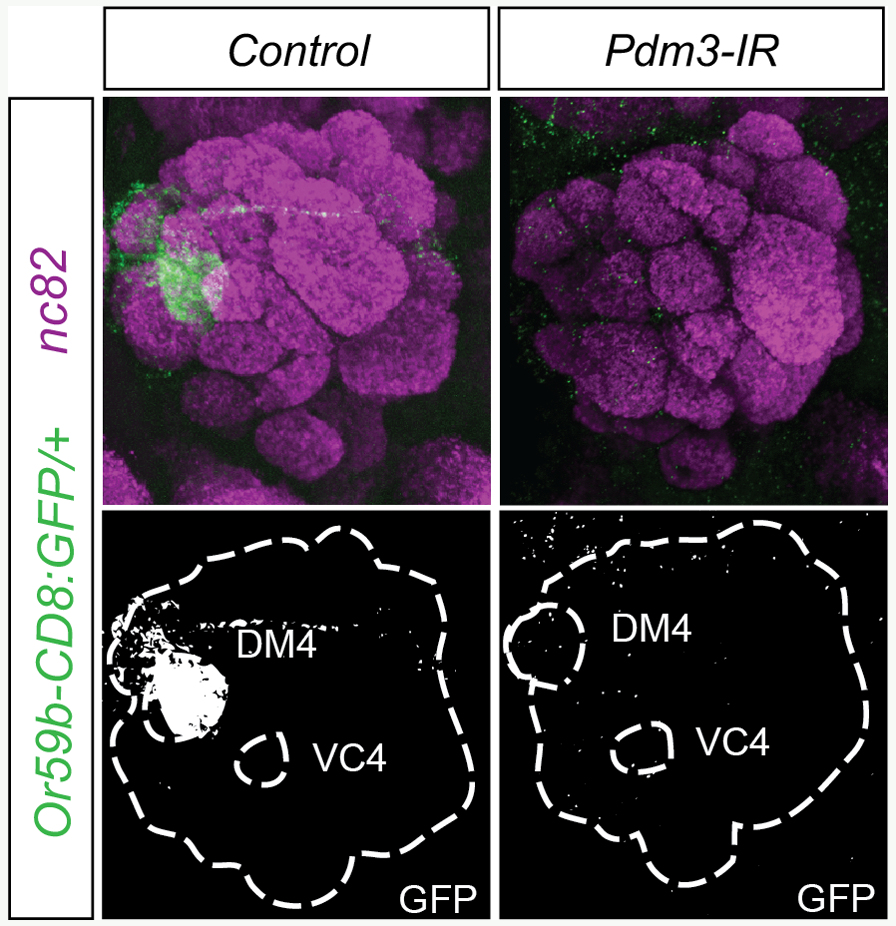

Supplement: S2 Fig — Pdm3 regulates Or59b expression. In Pdm3-knockdown flies, expression of the Or59b reporter is lost (GFP, green). Synaptic neuropil regions are labeled with the presynaptic marker nc82 (magenta). (TIF) [file pgen.1005051.s002.tif]

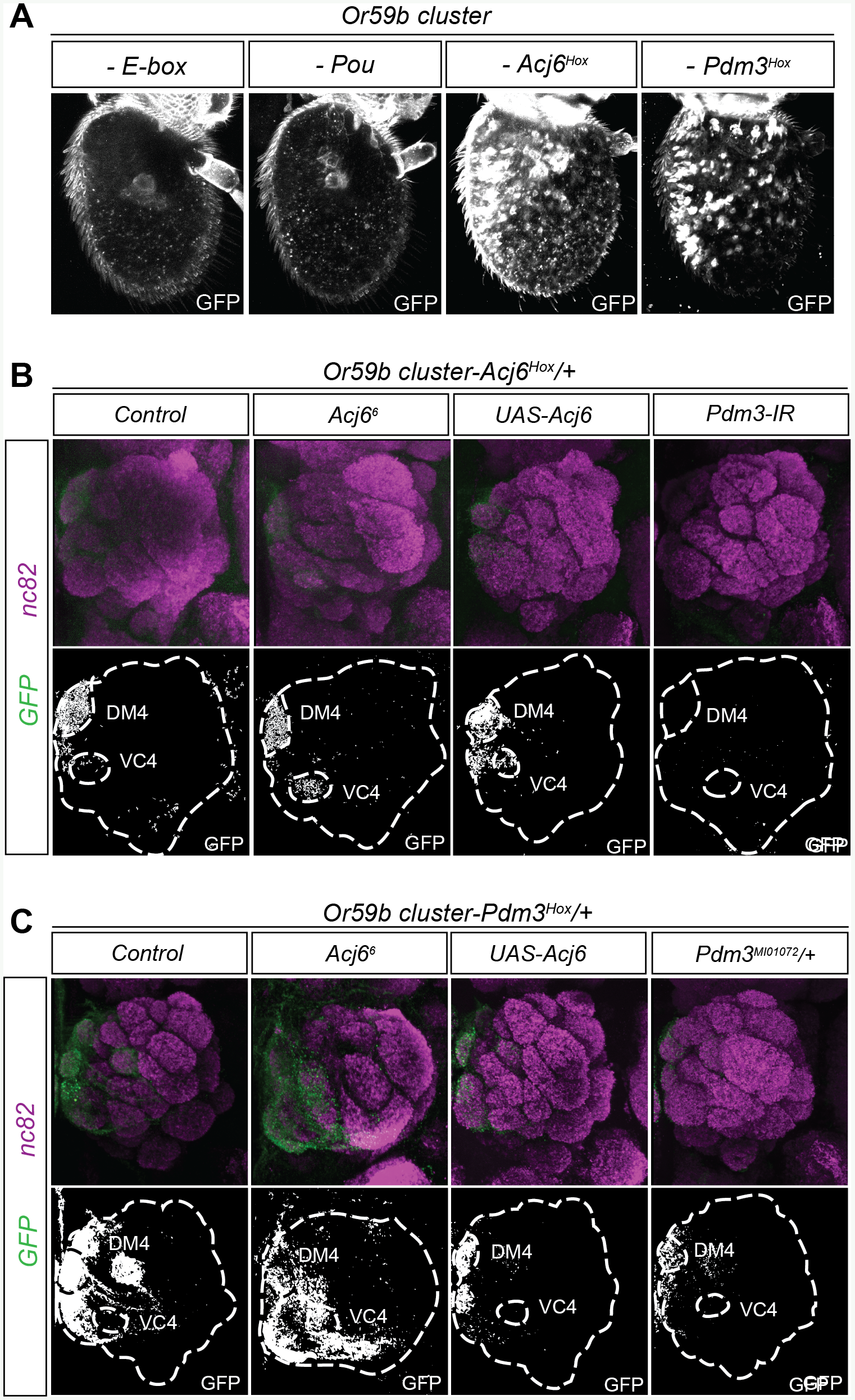

Supplement: S3 Fig — Epistasis experiments of Acj6 and Pdm3 regulation of the Or59b cluster. (A) Antennae that show the GFP expression produced by Or59b clusters with different mutations. (B) GFP expression (green) produced by an Acj6 Hox motif mutated cluster in different genetic backgrounds shows that the mutation made the cluster independent of Acj6 and placed pdm3 genetically downstream of acj6, as produced expression was lost in the Pdm3-IR flies. (C) GFP expression (green) produced by a Pdm3Hox motif-mutated cluster in different genetic backgrounds. The loss of ectopic expression in Pdm3 mutant heterozygote flies revealed that the auxiliary function of Acj6 is sufficient to support expression in the Ab2a and Ab7b classes. Overexpression of Acj6 attenuated the ectopic expression of the Or59b cluster, supporting a repressive function for both Acj6 and Pdm3. Synaptic neuropil regions are labeled with the presynaptic marker nc82 (magenta) in both B and C. (TIF) [file pgen.1005051.s003.tif]

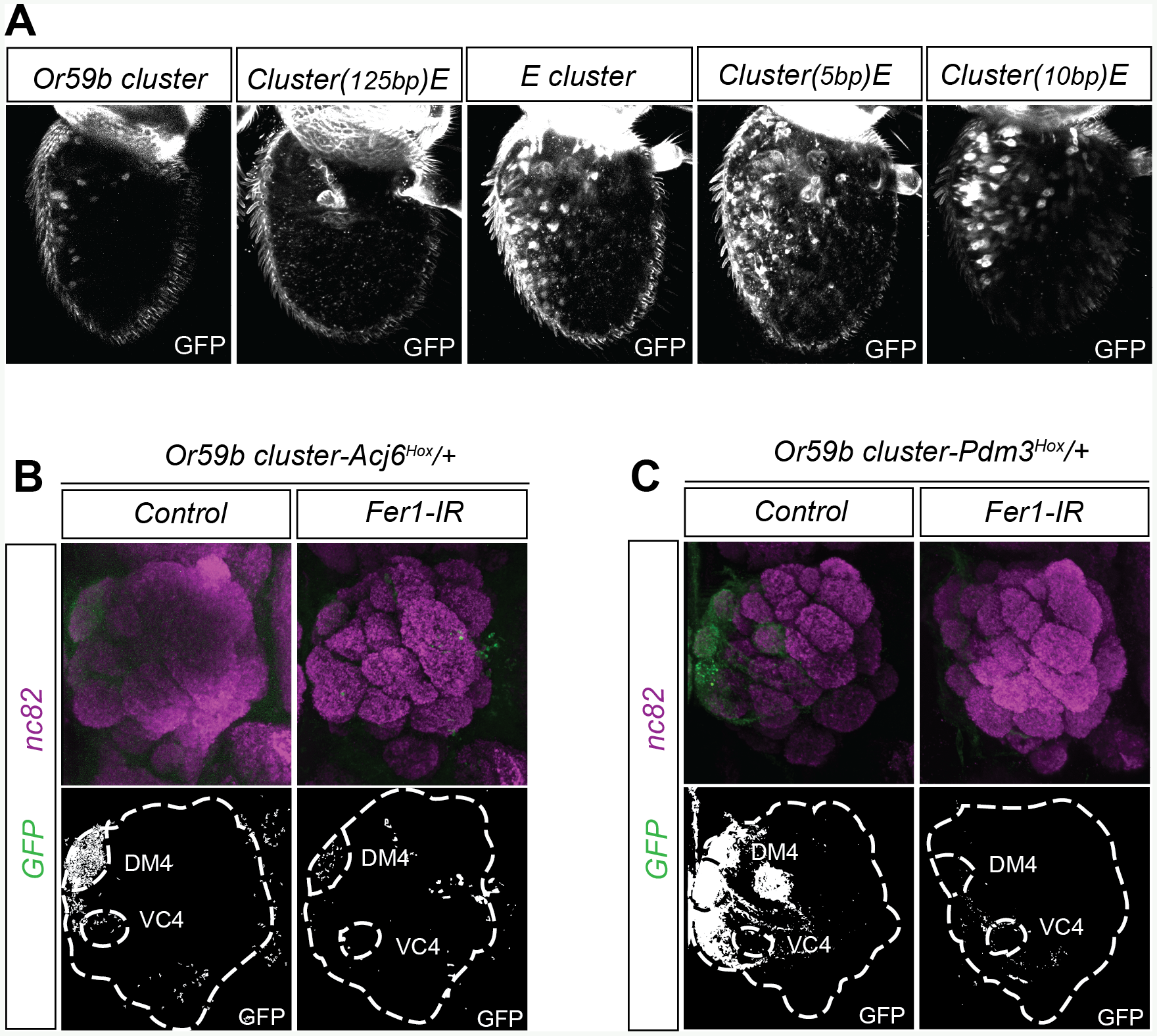

Supplement: S4 Fig — Genetic analysis of the Or59b cluster regulation by Fer1 and the E-box motif. (A) Antennae that show the GFP expression produced by Or59b clusters with a shifted or dislocated E-box. (B) GFP expression (green) produced by an Acj6 Hox motif mutated cluster. The partial but not full loss of expression in the Fer1-IR background indicates a weak redundant bHLH regulation repressed by Acj6. (C) GFP expression (green) produced by a Pdm3Hox motif mutated cluster. The total loss of ectopic GFP expression in the Fer1-IR background indicates that a combination of Pdm3 and Fer1 drives expression in the ectopic OSN classes and places Fer1 downstream of both Acj6 and Pdm3. (TIF) [file pgen.1005051.s004.tif]

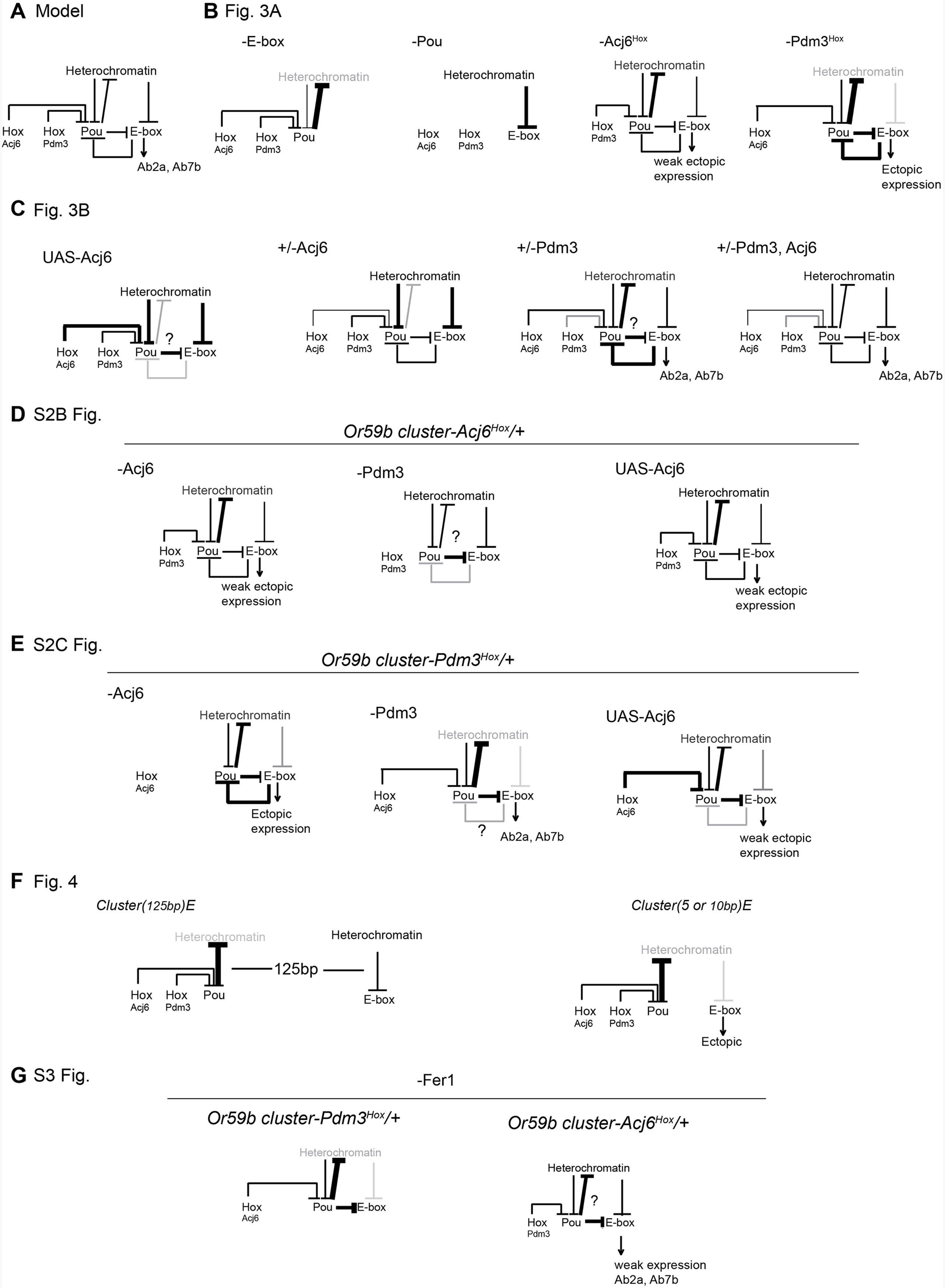

Supplement: S5 Fig — A model of Or59b cluster function and how the results of Figs. 3 and 4 can be predicted. (A) A model that summarizes the regulation events within the Or59b cluster. In short, the results show that Acj6 and Pdm3 bind two different Hox motifs and compete for a common Pou motif. We further show that the Hox interactions are repressive and that the Pou interaction is required to lift the suppression of the heterochromatin. We show that the E-box is downstream of Acj6, Pdm3 and the heterochromatin regulation. Our results further show that Pdm3 and Acj6 counteract the heterochromatin and likely allow binding to the E-box by bHLH proteins that in turn destabilize the POU interactions of Acj6 and Pdm3, establishing a steady state that supports expression in the Ab2a and Ab7b classes. (B-G) The models shown depict the predicted regulatory outcome of each genotype. Line thickness and blackness mark level of input (black, high input; light gray, low input). (TIF) [file pgen.1005051.s005.tif]

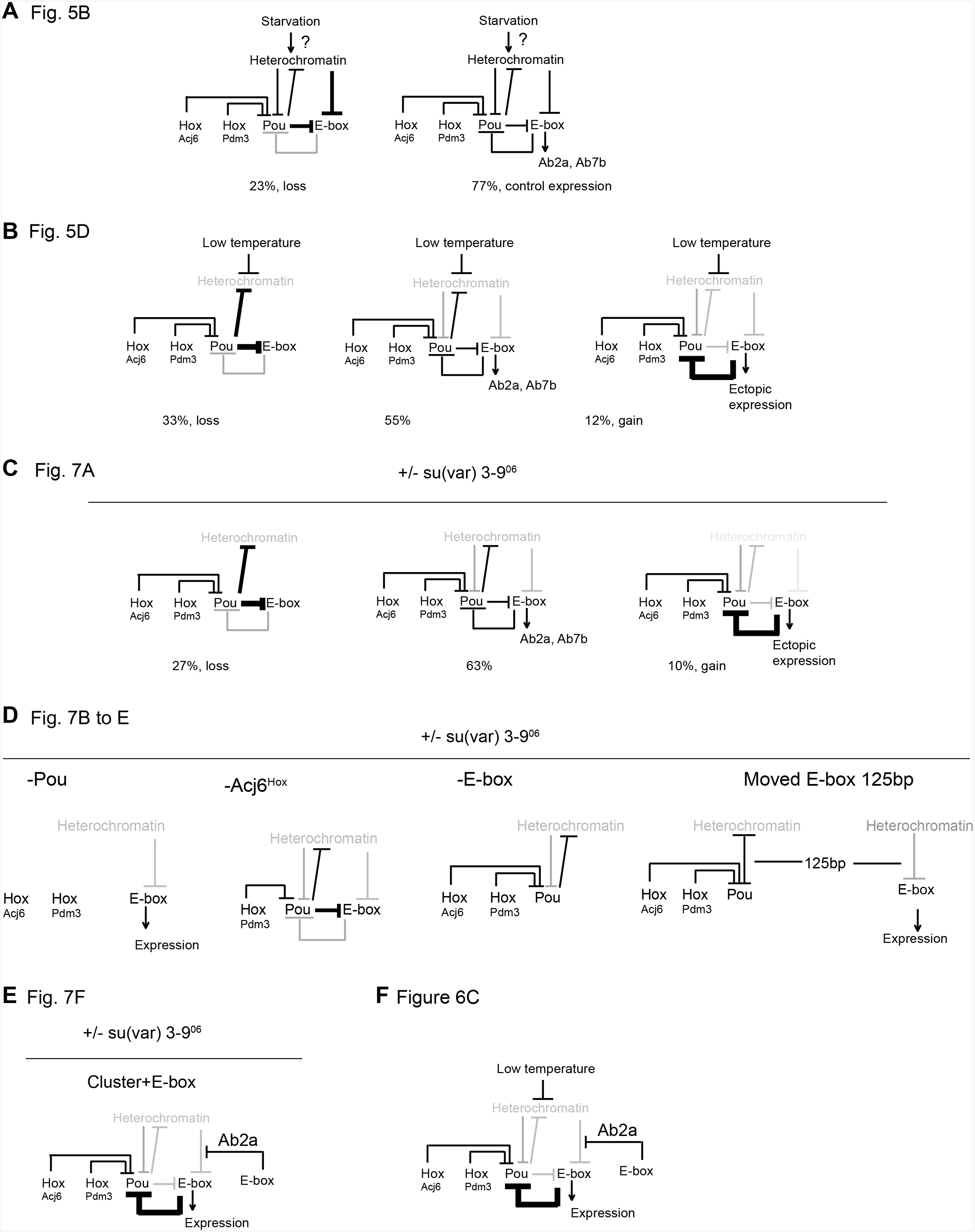

Supplement: S6 Fig — Models of the Or59b cluster modulation of environmental perturbations or Su(var)3–9 heterozygosity. (A-C) The models shown depict the predicted regulatory outcomes of each expression phenotype caused by starvation (A), temperature (B) and su(var)3–9 06 (reduced heterochromatin) (C). (D) The models shown depict the predicted interactions between the different mutant versions of the cluster and su(var)3–9 06. Reduced heterochromatin can generate increased binding at the Pou and E-box motifs. Rescue of the expression in the Pou mutant places the heterochromatin between the Pou and E-box and the output of the cluster downstream of the E-box. (E and F) The extra E-box stabilizes the expression of the Or59b cluster in su(var)3–9 heterozygous flies and in flies at low temperature, indicating that cooperative regulation supports specific OR expression. Line thickness and blackness indicate levels of input (black, high input; light gray, low input). (TIF) [file pgen.1005051.s006.tif]
