# Supplementary material for: Cis-Regulatory Mechanisms for Robust Olfactory Sensory Neuron Class-restricted Odorant Receptor Gene Expression in Drosophila
Source: PLoS Genet. 2015 Mar 11;11(3):e1005051. doi: 10.1371/journal.pgen.1005051 (PMC4356613; doi:10.1371/journal.pgen.1005051)
Supplement: S1 Table — Core motifs used in the bioinformatics analysis. (DOCX) [file pgen.1005051.s007.docx]

**Supplemental Table 1**

**Core motifs used in the bioinformatics analysis**

|  | Motif | % ORs with motif | average # motif/OR | |
| --- | --- | --- | --- | --- |
|  |  |  | regulated | non-regulated |
| E-box | CAXXTG | 96,3% | 5,0 | 4,53 |
| Onecut Hox | A**ATCAA** | 100% | 6,83 | 4,33 |
| Onecut Cut | ATCGAT | 100% | 6,17 | 4,95 |
| Acj6 Hox | CAATTAT | 100% | 6,88 | 5,50 |
| Acj6 Pou | TGCAT/A | 100% | 12,94 | 13,7 |

Core motif sequences used in bioinformatics search are mentioned. Each core motif was identified upstream of all analyzed ORs Except for E-box that was not found in the upstream region of Or19a.
