# Supplementary material for: Cis-Regulatory Mechanisms for Robust Olfactory Sensory Neuron Class-restricted Odorant Receptor Gene Expression in Drosophila
Source: PLoS Genet. 2015 Mar 11;11(3):e1005051. doi: 10.1371/journal.pgen.1005051 (PMC4356613; doi:10.1371/journal.pgen.1005051)
Supplement: S2 Table — List of transgenic flies lines. (DOCX) [file pgen.1005051.s008.docx]

| Transgene | Expressed insertions % | | | Total  # insertions | Comment |
| --- | --- | --- | --- | --- | --- |
|  | 0-20% | 20-80% | 80-100% |  |  |
| Or85a cluster* | 0 | 0 | 6 | 6 | Expressed |
| Or59b cluster (10768-1)** | 3 | 2 | 4 | 9 | Weak |
| Or59b cluster (13855-1)** | 5 | 2 | 3 | 10 | Weak |
| 2×Or19a Onecut (M7)* | 2 | 3 | 0 | 5 | Expressed |
| 2×Or19a One cut** | 1 | 1 | 2 | 4 | Expressed |
| 6×Or19a Onecut (M4)* | 5 | 4 | 1 | 10 | Expressed |
| 6×Or19a Onecut (M24)* | 2 | 2 | 3 | 7 | Expressed |
| 6×Or19a One cut** | 0 | 0 | 5 | 5 | Expressed |
| 2×Or85a Acj6 (12097-1)** | 10 | 0 | 0 | 10 | Not expressed |
| 2×Or59b E-box (12590-1)** | 6 | 0 | 0 | 6 | Not expressed |
| 2×Or19a Pdm3 (9579-3)** | 10 | 0 | 0 | 10 | Not expressed |
| E Or59b cluster (10768-5)** | 0 | 1 | 8 | 9 | Weak but ectopic |
| Or59b cluster (5bp) E (10768-2)** | 1 | 1 | 7 | 9 | Weak but ectopic |
| Or59b cluster (10bp) E (10768-3)** | 0 | 0 | 6 | 6 | Strong and ectopic |
| Or59b cluster (125bp) E (12290-5)** | 10 | 0 | 0 | 10 | Not expressed |
| Or59b cluster synthetic1 (SOF8)** | 5 | 0 | 0 | 5 | Not expressed |
| Or59b cluster synthetic2 (11585-1)** | 6 | 0 | 0 | 6 | Not expressed |
| Or59b cluster synthetic3 (11585-2)** | 8 | 0 | 0 | 8 | Not expressed |
| Or59b cluster synthetic4 (11585-3)** | 6 | 0 | 0 | 6 | Not expressed |
| Or59b cluster mutated E-box (M15)* | 6 | 0 | 0 | 6 | Not expressed |
| Or59b cluster mutated E-box (SOF 31)** | 3 | 0 | 0 | 3 | Not expressed |
| Or59b cluster mutated Pou (SOF32)** | 3 | 0 | 0 | 3 | Not expressed |
| Or59b cluster mutated Pdm3^Hox^ (SOF33)** | 0 | 0 | 3 | 3 | Strong and ectopic |
| Or59b cluster mutated Acj6^Hox^ (SOF34)** | 0 | 0 | 3 | 3 | Weak but ectopic |
| Or59b cluster+ E-box (10768-4)** | 4 | 2 | 3 | 9 | Expressed |
| Or59b cluster+ E-box (13855-2)** | 5 | 2 | 2 | 9 | Expressed |
| 2×Or59b cluster (6283-1)*** | 3 | 2 | 5 | 10 | Strong and ectopic |
| 2×Or59b cluster (M16)* | 4 | 1 | 2 | 7 | Strong and ectopic |
| 2×Or59b cluster (13855-3)** | 0 | 0 | 10 | 10 | Strong and ectopic |

**Supplemental Table 2, List of transgenic lines**

The number of insertions and their GFP expression status is presented. The percentages show the fraction of flies with detectable GFP expression. A total of 3-6 drosophila brains were dissected for each insertion. If GFP expression was detected in more than 30% of insertions it was considered as expressed.

*HSP core promoter

**Synthetic core promoter

***Eve core promoter
