# Supplementary material for: Cis-Regulatory Mechanisms for Robust Olfactory Sensory Neuron Class-restricted Odorant Receptor Gene Expression in Drosophila
Source: PLoS Genet. 2015 Mar 11;11(3):e1005051. doi: 10.1371/journal.pgen.1005051 (PMC4356613; doi:10.1371/journal.pgen.1005051)
Supplement: S3 Table — Summary of the environmental phenotypes. The table presents the fraction of the flies with mentioned phenotype in various metabolic and environmental conditions. (DOCX) [file pgen.1005051.s009.docx]

**Supplemental Table 3, summary of the environmental experiment phenotypes**

**Starvation**

***Or59b***

| Met Line |  | Food |  |  | Starvation |  |
| --- | --- | --- | --- | --- | --- | --- |
|  | Lost | Ectopic | Wild type | Lost | Ectopic | Wild type |
| II | 0/24 | 0/24 | 24/24 | 0/18 | 0/18 | 18/18 |
| III | 0/17 | 0/17 | 17/17 | 0/16 | 0/16 | 16/16 |

***1×59b cluster***

| Line |  | Food |  |  | Starvation |  |
| --- | --- | --- | --- | --- | --- | --- |
|  | Lost | Ectopic | Wild type | Lost | Ectopic | Wild type |
| 10768-1-1 | 0/25 | 0/25 | 25/25 | 7/28 | 0/28 | 21/28 |
| 10768-1-9 | 0/7 | 0/7 | 7/7 | 1/7 | 0/7 | 6/7 |

***1×59b cluster+E***

| Met Line |  | Food |  |  | Starvation |  |
| --- | --- | --- | --- | --- | --- | --- |
|  | Lost | Ectopic | Wild type | Lost | Ectopic | Wild type |
| 10768-4-5 | 0/27 | 0/27 | 27/27 | 0/25 | 0/25 | 25/25 |
| 10768-4-9 | 0/9 | 0/9 | 9/9 | 0/8 | 0/8 | 8/8 |

***Or85a cluster***

| Met Line |  | Food |  |  | Starvation |  |
| --- | --- | --- | --- | --- | --- | --- |
|  | Lost | Ectopic | Wild type | Lost | Ectopic | Wild type |
| M21-1 | 0/23 | 0/23 | 23/23 | 0/21 | 0/21 | 21/21 |
| M21-5 | 0/10 | 0/10 | 10/10 | 0/12 | 0/12 | 12/12 |
| M22-3 | 0/8 | 0/8 | 8/8 | 0/8 | 0/8 | 8/8 |

**Temperature**

***Or59b***

| Tm Line |  | 14⁰ |  |  | 24⁰ |  |  | 34⁰ |  |
| --- | --- | --- | --- | --- | --- | --- | --- | --- | --- |
|  | Lost | Ectopic | Wild type | Lost | Ectopic | Wild type | Lost | Ectopic | Wild type |
| II | 0/46 | 0/46 | 46/46 | 0/42 | 0/42 | 42/42 | 0/24 | 0/24 | 24/24 |
| III | 0/18 | 0/18 | 18/18 | 0/17 | 0/17 | 17/17 | 0/12 | 0/12 | 12/12 |

***1×59b cluster***

| Tm Line |  | 14⁰C |  |  | 24⁰C |  |  | 34⁰C |  |
| --- | --- | --- | --- | --- | --- | --- | --- | --- | --- |
|  | Lost | Ectopic | Wild type | Lost | Ectopic | Wild type | Lost | Ectopic | Wild type |
| 10768-1-1 | 16/45 | 8/45 | 21/45 | 0/41 | 0/41 | 41/41 | 0/15 | 0/15 | 15/15 |
| 10768-1-9 | 6/15 | 1/15 | 8/15 | 0/13 | 0/13 | 13/13 | 0/14 | 0/14 | 14/14 |
| 13855-1-1 | 3/18 | 0/18 | 15/18 | 0/20 | 0/20 | 20/20 | - | - | - |
| 13855-1-5 | 3/8 | 1/8 | 4/8 | 0/8 | 0/8 | 0/8 | - | - | - |
| 13855-1-8 | 4/10 | 2/10 | 4/10 | 0/9 | 0/9 | 9/9 | - | - | - |

***2×Or59b cluster***

| Tm Line |  | 14⁰C |  |  | 24⁰C |  |
| --- | --- | --- | --- | --- | --- | --- |
|  | Lost | Ectopic | Wild type | Lost | Ectopic | Wild type |
| 6283-2 | 1/11 | 2/11 | 8/11 | 0/11 | 0/11 | 11/11 |
| 6283-4 | 3/11 | 1/11 | 8/11 | 0/11 | 0/11 | 11/11 |
| 13855-3-1 | 5/16 | 2/16 | 9/16 | 0/16 | 0/16 | 16/16 |

***1×59b cluster+E***

| Tm Line |  | 14⁰C |  |  | 24⁰C |  |  | 34⁰C |  |
| --- | --- | --- | --- | --- | --- | --- | --- | --- | --- |
|  | Lost | Ectopic | Wild type | Lost | Ectopic | Wild type | Lost | Ectopic | Wild type |
| 10768-4-5 | 0/17 | 0/17 | 17/17 | 0/18 | 0/18 | 18/18 | 0/15 | 0/15 | 15/15 |
| 10768-4-9 | 0/15 | 0/15 | 15/15 | 0/15 | 0/15 | 15/15 | 0/11 | 0/11 | 14/11 |
| 13855-2-3 | 0/9 | 0/9 | 9/9 | 0/9 | 0/9 | 9/9 | - | - | - |

***Or85a cluster***

| Tm Line |  | 14⁰C |  |  | 24⁰C |  |  | 34⁰C |  |
| --- | --- | --- | --- | --- | --- | --- | --- | --- | --- |
|  | Lost | Ectopic | Wild type | Lost | Ectopic | Wild type | Lost | Ectopic | Wild type |
| M21-1 | 0/13 | 0/13 | 13/13 | 0/11 | 0/11 | 11/11 | 0/15 | 0/15 | 15/15 |
| M22-3 | 0/9 | 0/9 | 9/9 | 0/9 | 0/9 | 9/9 | 0/11 | 0/11 | 11/11 |
