# Supplementary material for: Cis-Regulatory Mechanisms for Robust Olfactory Sensory Neuron Class-restricted Odorant Receptor Gene Expression in Drosophila
Source: PLoS Genet. 2015 Mar 11;11(3):e1005051. doi: 10.1371/journal.pgen.1005051 (PMC4356613; doi:10.1371/journal.pgen.1005051)
Supplement: S4 Table — Primer sequences for the qPCR. (DOCX) [file pgen.1005051.s010.docx]

**Supplemental Table 4, Primers used in the qPCR analysis**

| Name | Forward primer (from 5' to 3') | Reverse primer (from 5' to 3') |
| --- | --- | --- |
| Or19a | ACGCCGTGCTGAAGAACTG | TTTGCGCCTCTTCAAGTCACT |
| Or22a | AACAAATTTGGCTATGGTGAAGCT | TTGAAACCTTTCGGCCAAGT |
| Or23a | AAGACTCTGGAGGAGAACGAACA | TCGAGTAATCTATACAGCGCGTTT |
| Or47b | GCTTTCCCCACCAATTTGATAA | TGATAAGAGGTTCCACGAGGAGTT |
| Or59b | GGACCAGCTATCCGACACGTA | ATGTGGGCCCGGAACATA |
| Or67c | CAATAGGATTTGCTGCACTGAAA | TGCCGCGAGCTTGAAAGT |
| Or67d | TCAAATGAGGATTTCCTAAGTGTG | ATTTTGAGCCTTGGCCAGC |
| Or85a | GAATGGAATACTCTTTGCAGAATGC | GGACGCAGAGTGTTTCCATATCT |
| Or92a | TGGACCTCAGCGAGGAGAAATGA | TAAATTGCAGGATGCGTTTG |
| Or98a | CAATCTCATTATTGACTATGCTGC | TGATCATTGAAAGGCCAAGG |

List of all Oligonucleotide sequences (5′ to 3′) used for RT-qPCR.
